# Quantification of Ethanedinitrile in Air Using a New and Accurate Gas Chromatography Method

**DOI:** 10.3390/mps2010001

**Published:** 2018-12-20

**Authors:** Sam E. Brierley, Anthony R. Adlam, Matthew K. D. Hall

**Affiliations:** 1 The New Zealand Institute for Plant & Food Research Limited, Private Bag 11600, Palmerston North 4442, New Zealand; sam.brierley@plantandfood.co.nz (S.E.B.); anthony.adlam@plantandfood.co.nz (A.R.A.); 2 Apreso, PO Box 2407, Palmerston North 4442, New Zealand

**Keywords:** cyanogen, EDN Fumigas™, quarantine, soil, Sterigas 1000™, method validation

## Abstract

Compared to previously tested fumigants such as methyl bromide, sulfuryl fluoride and phosphine; ethanedinitrile (EDN) is a new fumigant which is being trialled around the world as a pre-plant soil treatment and as a quarantine and pre-shipment (QPS) treatment of commodities. To collect the data necessary to assess the effectiveness of this fumigant, an accurate analytical method is needed across a wide concentration range. We reviewed the methods of detection for EDN described in recently published fumigation studies and have developed and validated a method to quantify EDN in air using a gas chromatograph equipped with a flame ionization detector (GC–FID). Our tested method has a linearity, precision, accuracy, limit of detection (LOD) and limit of quantification (LOQ) of R^2^ 0.9988, 1.36%, 98.8%, 0.750 ppm and 1.073 ppm, respectively. These values were determined using internationally recognised guidelines for the validation of non-standard analytical methods, which means that our method can be applied to the different validation requirements of regulatory agencies and countries. Our method can be used for experimental conditions that require detection at low and high concentrations simultaneously because it is accurate, fast (0.6 min) and repeatable across a concentration range of 1 to 40,000 ppm. This method will help to standardise the quantification of EDN by research groups and facilitate acceptance of data by regulatory organisations around the world.

## 1. Introduction

Fumigants are commonly used to disinfest commodities of insects and pathogens prior to export. There are several fumigants which have been extensively tested and are globally used as quarantine and pre-shipment (QPS) treatments of forest products, including methyl bromide, sulfuryl fluoride and phosphine which have been found to be highly effective against pests of logs and wood packaging material [1,2]. However, the continued use of some of these chemicals has negative environmental consequences as methyl bromide depletes the ozone and sulfuryl fluoride is a greenhouse gas [3]. The long-term future of methyl bromide and sulfuryl fluoride as QPS treatments of forest products is therefore uncertain [4].

Since these fumigants have been widely used in international trade for decades, validated analytical methods for their quantification in air have already been established [5]. Ethanedinitrile (EDN) is a new fumigant being trialled as a chemical disinfestation treatment to replace methyl bromide. It was recently registered in Australia as a treatment for logs and sawn timber moved between states [6], and became available for testing in New Zealand in 2011 [7]. Studies which have evaluated the toxicity of EDN to insect pests have used various analytical techniques (Table 1), however, to our knowledge, there does not exist a standardised technique for accurately quantifying EDN.

Recent studies have typically assessed the effectiveness of EDN in three core areas: (a) soil fumigation [8,9,10], (b) the treatment of fruits and vegetables [11], and (c) timber and logs [12,13,14]. Further development of EDN for these applications is currently focused on the treatment of soil and wood, as the use of EDN at low concentrations appears to be phytotoxic in fruits and vegetables [11]. As EDN is being trialled around the world as a new disinfestation treatment and as an alternative fumigant to methyl bromide for timber exports, a fast, accurate and repeatable method for its quantification is urgently required.

A significant advantage to using EDN as a fumigant is that, unlike the fumigants methyl bromide or sulfuryl fluoride, EDN is neither an atmospheric ozone depleting molecule nor is it a greenhouse gas [15,16].

The mode of action for insects is thought to be like that of other inorganic cyanides, whereby EDN reduces to cyanide which interrupts the cytochrome c oxidase complex within the organism [17]. This leads to the inability to transport oxygen throughout the body, resulting in respiratory inhibition and ultimately asphyxiation or suffocation.

Quantifying the concentration of EDN in air may seem straightforward, as an EDN flame burns at 4,525 °C [18]. Hence, a number of analytical techniques have been used, of which gas chromatograph with a flame ionization detector (GC–FID) is the most common (Table 1).

Of the twelve most recently published studies relating to fumigation science and the measurement of EDN, eight of them used a GC–FID method, and four used a gas chromatograph with a nitrogen phosphorus detector (GC–NPD) or a fumigant monitor fitted with a thermal conductivity detector (TCD) (Table 1). The columns used for separation with the most common GC–FID method are HP5, GS–Q and DB–WAX (Table 1), which are all general-purpose columns for the separation of a wide range of analytes. Four out of the eight GC–FID methods have used a GS–Q column for the separation of EDN. Therefore, the most common method of quantifying EDN in air for fumigation research is with GC–FID using a GS–Q column. This is a porous polymer, fused silica PLOT (porous layer open tubular) column and is designed for the separation of smaller molecules, such as EDN.

The aim of our study was to propose and validate an accurate, fast and repeatable analytical method to quantify EDN across a wide range of concentrations that would support fumigant research and ensure the integrity of analytical data. In addition, regulatory agencies require validation of any non-standard analytical method to accept physicochemical, toxicological and ecotoxicological data supplied as part of the chemical registration process. We provide here the data necessary to validate a method for the quantification of EDN in air that can be used universally and the results accepted internationally. 

## 2. Materials and Methods

### 2.1. Preparation of Samples

The EDN Fumigas^TM^ used in these tests was drawn from stocks held at the Plant & Food Research disinfestation laboratory (Palmerston North, New Zealand). The Manufacturer’s Certificate of Analysis (CoA) for the EDN used certified that the cylinders contained 98.32% EDN, 0.25% hydrogen cyanide and other unspecified impurities. EDN was dispensed from a high-pressure cylinder into a 20-L Tedlar^®^ bag (SKC Ltd., Dorset, UK). Concentrations of EDN were then prepared in 1-L Tedlar bags using airtight gas syringes (Hamilton^®^, Reno, Nevada, NV, USA), a 3 mL sample was then injected into the GC with a 250 µL sample loop (Table 2).

### 2.2. Analytical Conditions and Equipment

Gas samples were analysed by gas chromatography using an Agilent 7890A (Santa Clara, CA, USA) equipped with a FID. The conditions under which the method was validated are described in Table 2. Peak integration was performed using ChemStation software, Agilent Technologies (Sanata Clara, CA, USA).

### 2.3. Validation Guidelines

To validate our method we followed the guidelines outlined by the International Council for Harmonisation of Technical Requirements for Pharmaceuticals for Human Use (ICH) [27], European Commission-Technical Materials and preparations (EC) [28] and the Australian Pesticides and Veterinary Medicines Authority (APVMA) [29]. These guidelines are recognised methods for the validation of non-standard analytical methods. The calculation of parameters from these guidelines include, but are not exclusive to, linearity, precision, accuracy, limit of detection (LOD) and limit of quantification (LOQ).

### 2.4. Linearity

The ability to produce test results that are proportional to the concentration of the analyte in samples must be tested within 80–120% of the anticipated concentration range. A correlation coefficient (R^2^) of ≥0.99 must be achieved with a linear response across 6–8 concentrations for the method to meet the linearity criterion. Linearity was determined across seven concentrations (0, 5,000, 10,000, 15,000, 20,000, 25,000 and 40,000 ppm) of EDN in air. The average response of the instrument to five replicates at each concentration was determined.

### 2.5. Precision

Precision was assessed by measuring the repeatability of the instrument across six concentrations of EDN in air that are typically used for fumigation research, with five replicates per concentration. Precision was measured by calculating the standard deviation (SD), percent standard error (%SE) and percent relative standard deviation (%RSD) of each concentration relative to the average. An average precision of ≤2% must be achieved to meet the ICH, EC and APVMA guidelines.

### 2.6. Accuracy

Accuracy is expressed as the degree to which the determined value of an analyte in a sample corresponds to a true value. To evaluate the accuracy of a method, guidelines require that a mean recovery of 98–102% is achieved.

### 2.7. Limit of Detection

The LOD is the lowest amount of an analyte that can be detected reliably against a blank sample, but not necessarily quantified as an exact value. The LOD or detection limit (DL) is calculated differently for ICH and APVMA guidelines, and both approaches were used (Table 3). EC guidelines do not clearly define how the LOD should be calculated.

### 2.8. Limit of Quantification

The LOQ or quantification limit (QL) is the lowest concentration of an analyte that can be quantified in a sample and was calculated using ICH and APVMA guidelines (Table 4); while the EC guidelines do not state how LOQ is to be calculated.

## 3. Results

### 3.1. Linearity

The linearity of EDN was determined across seven concentrations (0, 5000, 10,000, 15,000, 20,000, 25,000 and 40,000 ppm) using the average of five replicates at each concentration. The response of the instrument was linear with an R^2^ value of 0.9988 (y = 0.3693x)), indicating that the linearity of this method passed all of the guidelines [2,3,4,5,6,7,8,9].

### 3.2. Precision

Precision of the method was determined by the analysis of six concentrations ranging from 25 to 200% of the expected concentration range. The response of the instrument under the conditions tested is summarised in Table 5.

The average RSD across the concentration range tested was 1.36% (Table 5), which meets the requirement of ≤2% defined by the ICH and APVMA guidelines for precision [27,29].

### 3.3. Accuracy

Accuracy was measured by establishing three concentrations (15,000, 20,000 and 25,000 ppm) that correspond to between 80 and 120% of the expected concentration range and collecting five replicate samples of each concentration.

The average accuracy of the GC–FID method was calculated by combining the average response of five replicates across three concentrations. The average accuracy of the method under the conditions tested was 98.8% (Table 6). This meets the accuracy requirement of 98–102% specified by respective guidelines [27,28,29].

### 3.4. Limit of Detection and Limit of Quantification

The LOD and LOQ of the method were determined by repeated measurements of the lowest repeatable concentration of EDN at 10 different instances (Table 7). The average measured concentration and %RSD are presented in Table 7. With the formulae presented in Table 3 and Table 4, these data were used to calculate the LOD and LOQ of the method.

#### 3.4.1. LOD Calculation

Using the formulae in Table 3 and the data in Table 7, the LOD was 0.750 and 0.138 ppm using the APVMA and the ICH guidelines, respectively.

#### 3.4.2. LOQ Calculation

Using the formulae in Table 4 and data in Table 7, the LOQ was 1.073 and 0.461 ppm using the APVMA and the ICH guidelines, respectively.

**Figure 1:**
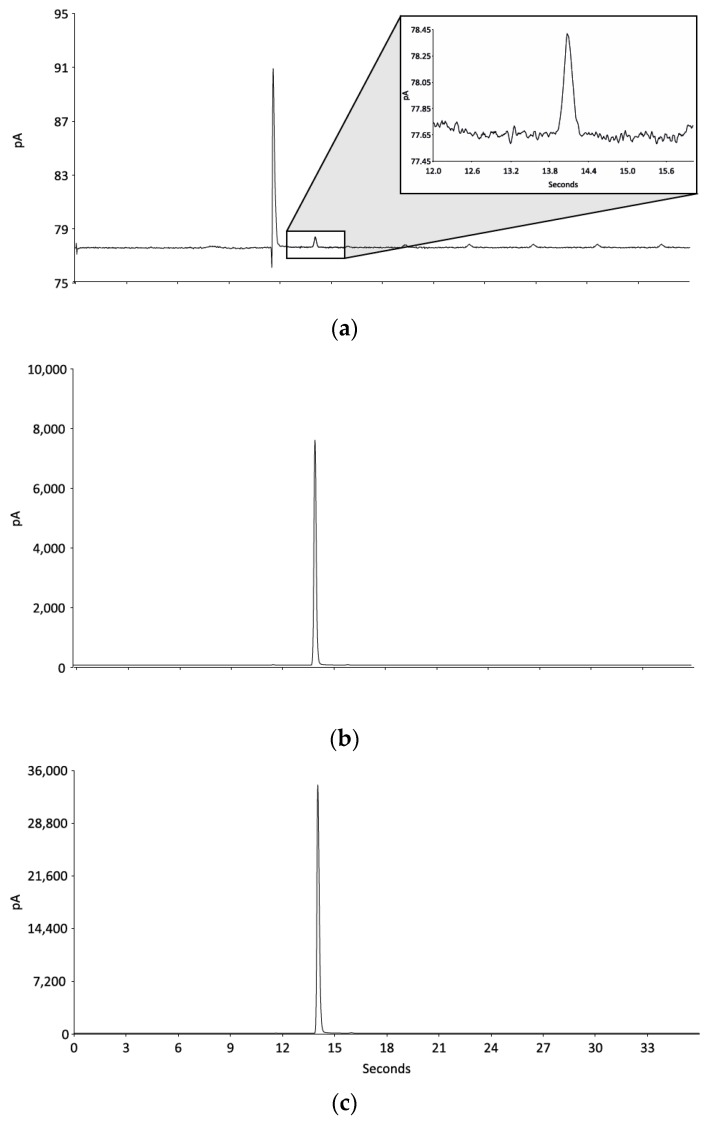
Chromatograms for three different concentrations of ethanedinitrile (**a**) low: 0.6 ppm, (**b**) medium: 5,000 ppm and (**c**) high: 25,000 ppm and their relative intensities.

The chromatograms in Figure 1 display the typical response for different concentrations of EDN. No other eluents are seen later in the run for high concentrations although a back flash of EDN is seen following the initial peak. This is common with methods that use a high flow rate and a faster run time [30]. This anomaly was validated as a back flash and not as a later eluent from a previous run (data not shown). Concentrations are calculated from the area under the curve by using defined cardinal points divided by the slope of the calibration curve. 

## 4. Discussion

Research activities with EDN that produce physicochemical, toxicological and ecotoxicological data must meet the requirements of regulatory agencies of different countries. An important step in this process is the validation of non-standard analytical methods that use guidelines which specify the minimum requirements across a range of factors to determine the quality of the method.

EDN is a relatively new fumigant that is being trialled around the world as a disinfestation treatment of soil and wood products prior to commercialisation. There is a range of analytical methods currently being used to measure EDN across research groups, with differences in the detection method and column used for separation (Table 1). Because the most common method of detection in published literature uses GC–FID with a GS–Q column, we have developed and validated an analytical method to measure EDN using this equipment.

Here we present and validate an accurate, fast and repeatable method for the quantification of EDN in air. Acceptable values for the parameters (linearity, precision, accuracy, LOD and LOQ) of three internationally accepted guidelines [27,28,29] were met. Unfortunately, comparisons between our method and those used by other studies are not possible, as this is the first time that a validated methodology to quantify the concentration of EDN in air has been proposed. It was not the focus of this work to test field collected samples, however, a number of studies [12,14,21] have used the same method and shown that it performs very well under these conditions. These studies did not go the extent of validating the method, as this work has done, but they indicate that the method is not affected by the coelution of other compounds during simulated commercial fumigations.

## 5. Conclusion

Our method is suitable for all EDN fumigation studies that require detection at low and high concentrations simultaneously. Our hope is that this method is used by researchers to standardise the way in which EDN is quantified during and after fumigation to facilitate the acceptance of data by regulatory authorities around the world.

## Figures and Tables

**Table 1:** List of recent studies (most recent to oldest) which quantify ethanedinitrile in air and their respective detection methods, columns and concentration ranges.

Authors	Detection Method	Column	Concentration Range (ppm) ^a^
**Lee et al.** [13]	GC–FID ^b^	HP–5	0–20,000
**Najar-Rodriguez et al.** [14]	GC–FID	GS–Q	0–25,000
**Hall et al.** [12]	GC–FID	GS–Q	0–25,000
Emery et al. [19]	GC–FID	GS–Q	0–20,000
Park et al. [20]	GC–FID	HP–5	0–75,000
Pranamornkith et al. [21]	GC–FID	GS–Q	0–25,000
Ren et al. [2]	GC–NPD ^c^	GS–Q	0–25,000
Cho et al. [22]	GC–FID	DB–WAX	0–40,000
Park et al. [23]	GC–FID	DB–WAX	0–20,000
Ren et al. [24]	XK–3–TCD ^d^	–	0–10,000
Sarwar et al. [25]	GC–NPD ^e^	J&W	0–50,000
O’Brien et al. [26]	GC–NPD ^e^	DB–WAX	0–20,000

^a^ Unless stated within the publication(s), the concentration range is the minimum and maximum recordings observed

^b^ GC–FID, gas chromatograph with a flame ionization detector

^c^ GC–NPD, gas chromatograph with a nitrogen phosphorus detector

^d^ XK–3–TCD, XK–3 fumigant monitor with a thermal conductivity detector

^e^ Reported as GC–TSD, gas chromatograph with thermionic sensitive detector also known as NPD.

**Table 2:** Instrument and conditions under which the proposed method for quantifying ethanedinitrile in air was validated.

Variable	Parameter
Laboratory temperature	25 ± 1 °C
Column	Agilent J&W GS–Q
Column dimensions	Length 30 m, internal diameter 0.53 mm, film thickness 0 mm
Carrier gas	Helium
Pressure	27 psi
Total flow	239.79 mL/min
Injection volume (sample loop volume)	3 mL (250 µL)
Split ratio	5:1 @ 197.32 mL/min
Temperature program	Isothermal 150 °C
Detector temperature	300 °C
Inlet temperature	150 °C
H_2_ flow	100 mL/min
Air flow	400 mL/min
Makeup flow (nitrogen)	0.5 mL/min
Total runtime	0.6 min

**Table 3:** Accepted methods for calculating the limit of detection (LOD) or detection limit (DL).

APVMA ^a^	ICH ^b^
The LOD of an analytical method is the lowest amount of an analyte in a sample that can be detected, but not necessarily quantified as an exact value.	The DL of an individual analytical procedure is the lowest amount of analyte in a sample which can be detected but not necessarily quantified as an exact value.
LOD=X+(3SD)	$\mathrm{DL}=\;\frac{3.3\;\mathrm{SD}}{b}$
X = Average response SD = The standard deviation of the response	b = slope of the calibration curve SD = standard deviation of the response

^a^ Australian Pesticides and Veterinary Medicines Authority (APVMA)

^b^ International Council for Harmonisation (ICH) of Technical Requirements for Pharmaceuticals for Human Use

**Table 4:** Accepted methods for calculating the limit of quantification (LOQ) or quantification limit (QL).

APVMA ^a^	ICH ^b^	EC ^c^
The limit of quantification (LOQ) is the lowest amount of the analyte in the sample that can be quantitatively determined with defined precision under the stated experimental conditions.	The quantification limit (QL) of an individual analytical procedure is the lowest amount of analyte in a sample which can be quantitatively determined with suitable precision and accuracy.	Defined as the lowest concentration tested at which an acceptable mean recovery with an acceptable RSD is obtained.
LOD=X+(10SD)	$\mathrm{QL}=\;\frac{10\;\mathrm{SD}}{b}$	** Not given
X = Average response SD = The standard deviation of the response	B = slope of the calibration curve SD = standard deviation of the response	

^a^ Australian Pesticides and Veterinary Medicines Authority (APVMA)

^b^ International Council for Harmonisation (ICH) of Technical Requirements for Pharmaceuticals for Human Use

^c^ European Commission (EC) Technical Material and Preparations

**Table 5:** Response of the instrument to different concentrations of ethanedinitrile to determine linearity and precision of the analytical method.

	Peak Area							
Conc. (ppm)	Rep. 1	Rep. 2	Rep. 3	Rep. 4	Rep. 5	Average	%SE ^a^	%RSD ^b^
5000	2024	2010	2084	2154	2105	2075.19	1.146	2.56
10,000	3988	4038	3932	3966	3976	3979.95	0.389	0.87
15,000	5459	5308	5390	5361	5447	5392.94	0.463	1.03
20,000	7095	7291	7319	7368	7284	7271.45	0.573	1.28
25,000	9383	9133	9180	9391	9365	9290.39	0.533	1.19
40,000	14,527	14,719	14,735	15,034	14,723	14,747.40	0.493	1.10
Average	–	–	–	–	–	–	0.607	1.36

^a^ Percentage standard error of the peak area

^b^ Percentage relative standard deviation

**Table 6:** Measured peak area of the instrument to different concentrations of ethanedinitrile to determine accuracy of the method.

Conc. (ppm)	Rep. 1	Rep. 2	Rep. 3	Rep. 4	Rep. 5	Average	Conc. (ppm) ^a^	Accuracy ^b^
15,000	5459	5308	5390	5361	5447	5392.943	14,603.31	97.4
20,000	7095	7291	7319	7368	7284	7271.45	19,690.04	98.5
25,000	9383	9133	9180	9391	9365	9290.393	25,157.05	100.6
Average	–	–	–	–	–	–	–	98.8

^a^ Concentration calculated from standard curve equation, calculated concentration (ppm)

^b^ Accuracy was calculated as a ratio of the calculated concentration/concentration to give a precision percentage

**Table 7:** Data used to calculate the limit of detection and limit of quantification of a GC–FID method used to measure ethanedinitrile in air.

Rep.	Conc. (ppm)
1	0.636
2	0.565
3	0.693
4	0.566
5	0.597
6	0.542
7	0.620
8	0.659
9	0.653
10	0.583
Average	0.611
SD ^a^	0.046
%RSD ^b^	7.543

^a^ Standard deviation

^b^ Percentage relative standard deviation
